# Analysis of nucleotide insertion opposite urea and translesion synthesis across urea by DNA polymerases

**DOI:** 10.1186/s41021-022-00236-3

**Published:** 2022-02-15

**Authors:** Taishu Kawada, Katsuhito Kino, Kyousuke Tokorodani, Ryuto Anabuki, Masayuki Morikawa, Takanobu Kobayashi, Kazuaki Ohara, Takayuki Ohshima, Hiroshi Miyazawa

**Affiliations:** 1 Kagawa School of Pharmaceutical Sciences, Tokushima Bunri University, 1314-1 Shido, Sanuki, Kagawa 769-2193 Japan; 2 Faculty of Science and Engineering, Tokushima Bunri University, 1314-1 Shido, Sanuki, Kagawa 769-2193 Japan

**Keywords:** Oxidative DNA damage, Urea, DNA polymerase, Base pair, Nucleotide incorporation, Elongation

## Abstract

**Abstract:**

Urea (Ua) is produced in DNA as the result of oxidative damage to thymine and guanine. It was previously reported that Klenow fragment (Kf) exo^−^ incorporated dATP opposite Ua, and that DNA polymerase β was blocked by Ua. We report here the following nucleotide incorporations opposite Ua by various DNA polymerases: DNA polymerase α, dATP and dGTP (dATP > dGTP); DNA polymerase δ, dATP; DNA polymerase ζ, dATP; Kf exo^−^, dATP; *Sulfolobus solfataricus* P2 DNA polymerase IV (Dpo4), dGTP and dATP (dGTP > dATP); and DNA polymerase η, dCTP, dGTP, dATP, and dTTP (dCTP > dGTP > dATP > dTTP). DNA polymerases β and ε were blocked by Ua. Elongation by DNA polymerases δ and ζ stopped after inserting dATP opposite Ua. Importantly, the elongation efficiency to full-length beyond Ua using DNA polymerase η and Dpo4 were almost the same as that of natural DNA.

**Graphical abstract:**

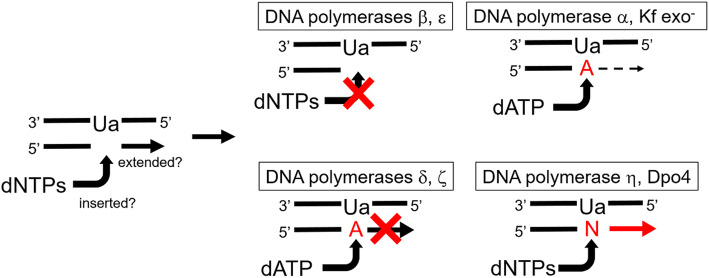

**Supplementary Information:**

The online version contains supplementary material available at 10.1186/s41021-022-00236-3.

## Introduction

**Fig. 1:**
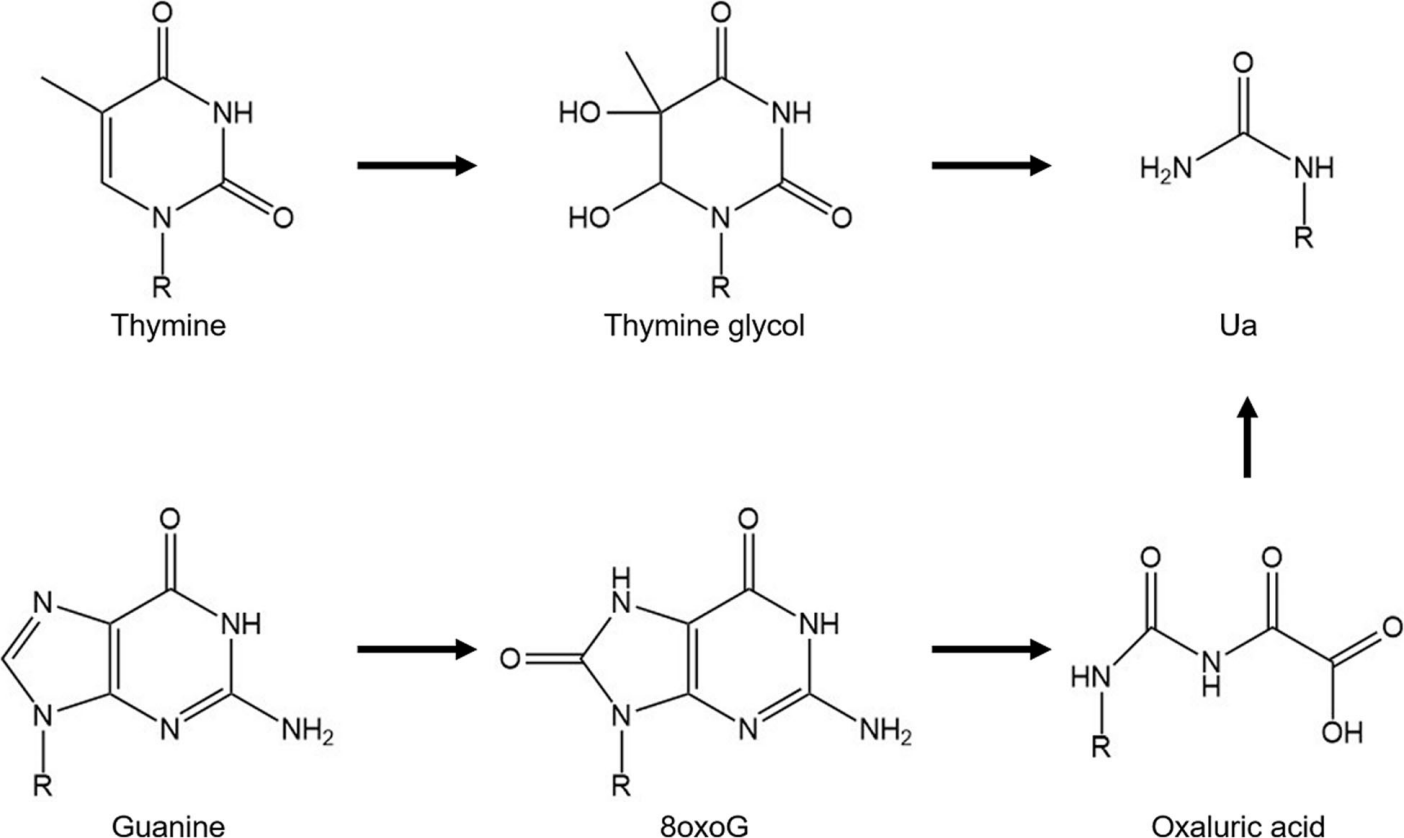
Generation of urea (Ua)

DNA damage is a major cause of cell death, mutations, cancer, neurological disease, and aging. Urea (Ua) is produced in DNA by ionizing radiation [1] due to oxidative damage to thymine [2–7] and guanine [8, 9] (Fig. 1). Although Ua is reportedly repaired by many enzymes [3, 5, 10–24], to date there have been only a few investigations into nucleotide incorporation and/or elongation past Ua by DNA polymerases, such as the blockage by Ua of DNA replication by T4 DNA polymerase [4, 25, 26], Klenow fragment (Kf) [4, 25–28], DNA polymerase I [25], human DNA polymerase β [28], Sequenase 2.0 [28], and AMV reverse transcriptase [28]. However, nucleotide incorporations opposite Ua and translesion synthesis across Ua by DNA polymerases α, δ, ε, η, ζ and *Sulfolobus solfataricus* P2 DNA polymerase IV (Dpo4) have not been analyzed to date. Herein, we describe our studies of nucleotide incorporations and translesion synthesis in relation to Ua with DNA polymerases α, δ, ε, η, ζ and Dpo4. Also, we confirmed nucleotide incorporation and extension in relation to Ua using Kf exo^−^ and DNA polymerase β.

## Materials and methods

### Enzymes

T4 polynucleotide kinase was purchased from New England Biolabs (Ipswich, USA). T4 DNA ligase was purchased from Takara (Otsu, Japan). Calf thymus DNA polymerase α and human DNA polymerase β were purchased from Chimerx (Milwaukee, USA). Klenow fragment exonuclease^−^ was purchased from Fermentas (Waltham, USA). Yeast DNA polymerase ζ was purchased from Enzymax (Lexington, USA). Human DNA polymerase δ [29], human DNA polymerase η [30], and *Saccharomyces cerevisiae* DNA polymerase ε [31] were purified as described previously. Dpo4 was purchased from Trevigen (Gaithersburg, USA).

### DNA substrates

The DNA template (5′-CTCATCAACATCTTXAATTCACAATCAATA-3′, X represents guanine), Alexa 680-labeled 15-mer primer (5′-Alexa680-TATTGATTGTGAATT-3′), 6-mer oligonucleotide containing 8oxoG (5′-CTT8oxoGAA-3′), 13-mer oligonucleotide (5′-TTCACAATCAATA-3′), and 11-mer oligonucleotide (5′-CTCATCAACAT-3′) were constructed by Japan Bio Services Co., Ltd. (Saitama, Japan).

The 30-mer DNA template containing Ua (30-merUa) (5′-CTCATCAACATCTTXAATTCACAATCAATA-3′, where X represents Ua) was constructed as follows. A 6-mer oligonucleotide containing 8oxoG (5′-CTT8oxoGAA-3′) was oxidized to a 6-mer oligonucleotide containing oxaluric acid (Oxa) using I_2_ and KI [32], then Oxa was hydrolyzed to provide a 6-mer oligonucleotide containing Ua (6-merUa) (5′-CTTXAA-3′, where X represents Ua) [9]. This 6-merUa and a 13-mer oligonucleotide (5′-TTCACAATCAATA-3′) were phosphorylated by T4 polynucleotide kinase. The 30-merUa was prepared by ligation of an 11-mer oligonucleotide (5′-CTCATCAACAT-3′) to the 5′ side of the 6-merUa and the phosphorylated 13-mer oligonucleotide to the 3′ side of the 6-merUa using T4 DNA ligase, using a 30-mer DNA-RNA chimeric oligonucleotide (5′-TATTGATTgTGAATTGCAGATgTTGATGAG-3′, where g represents guanosine, not deoxyguanosine) as a template. 30-merUa was isolated by HPLC (Fig. S1).

### Polymerization reaction

Polymerization reactions (5 μL) were carried out using the following mixtures: (for DNA polymerase α) 40 mM Tris-HCl (pH 8.0), 5 mM MgCl_2_, 10 mM NaCl, 45 mM KCl, 1 mM DTT, 100 μg/mL bovine serum albumin (BSA); (for DNA polymerase β) 50 mM Tris-HCl (pH 8.8), 10 mM MgCl_2_, 1 mM DTT, 100 μg/mL BSA; (for DNA polymerase δ) 50 mM Tris-HCl (pH 7.4), 2 mM MgCl_2_, 2 mM DTT, 100 μg/mL BSA; (for DNA polymerase ε) 50 mM Tris-HCl (pH 7.4), 8 mM MgCl_2_, 2 mM DTT, 100 μg/mL BSA; (for DNA polymerases ζ and η) 50 mM Tris-HCl (pH 8.0), 2 mM MgCl_2_, 5 mM DTT, 100 μg/mL BSA; (for Kf exo^−^) 50 mM Tris-HCl (pH 8.0), 5 mM MgCl_2_, 1 mM DTT, 100 μg/mL BSA; (for Dpo4) 20 mM HEPES (pH 6.5), 10 mM MgCl_2_, 1 mM MnCl_2_ 100 mM NaCl, 100 μg/mL BSA.

The reaction mixtures for DNA polymerases α, β, δ, ε, η, ζ, Kf exo^−^ and Dpo4 contained 100 fmol of the template and 50 fmol of 5′-Alexa680-labeled 15-mer primer. Other conditions and the concentrations of dNTPs and DNA polymerases are specified in the figure legends. Reactions were performed at 30 °C for 30 min for DNA polymerases α, β, δ, ε, ζ, Kf exo^−^ and Dpo4, and at 37 °C for 30 min for DNA polymerase η. All reactions were stopped by adding 5 μL of stop buffer (15 mM EDTA, 10% glycerol (v/v), and 100 μM rhodamine 6G). Aliquots (2.5 μL) were subjected to electrophoresis in a denaturing 16% polyacrylamide (v/v) gel containing 8 M urea at 30 W for 90 min. The fluorescence intensity of each n-mer band (*I*_n_) was quantified using an Odyssey infrared imaging system from LI-COR (Lincoln, USA) for 5′-Alexa 680-labeled products. The nucleotide incorporation efficiency was calculated using the formula: Σ *I*_n_ (*n* ≥ 16) / Σ *I*_n_ (*n* ≥ 15). The DNA synthesis efficiency was calculated using the formula: *I*_30_ / Σ *I*_n_ (*n* ≥ 15).

## Results

### Incorporation and translesion synthesis opposite Ua by DNA polymerases α, β, δ, ε and Kf exo^−^

**Fig. 2:**
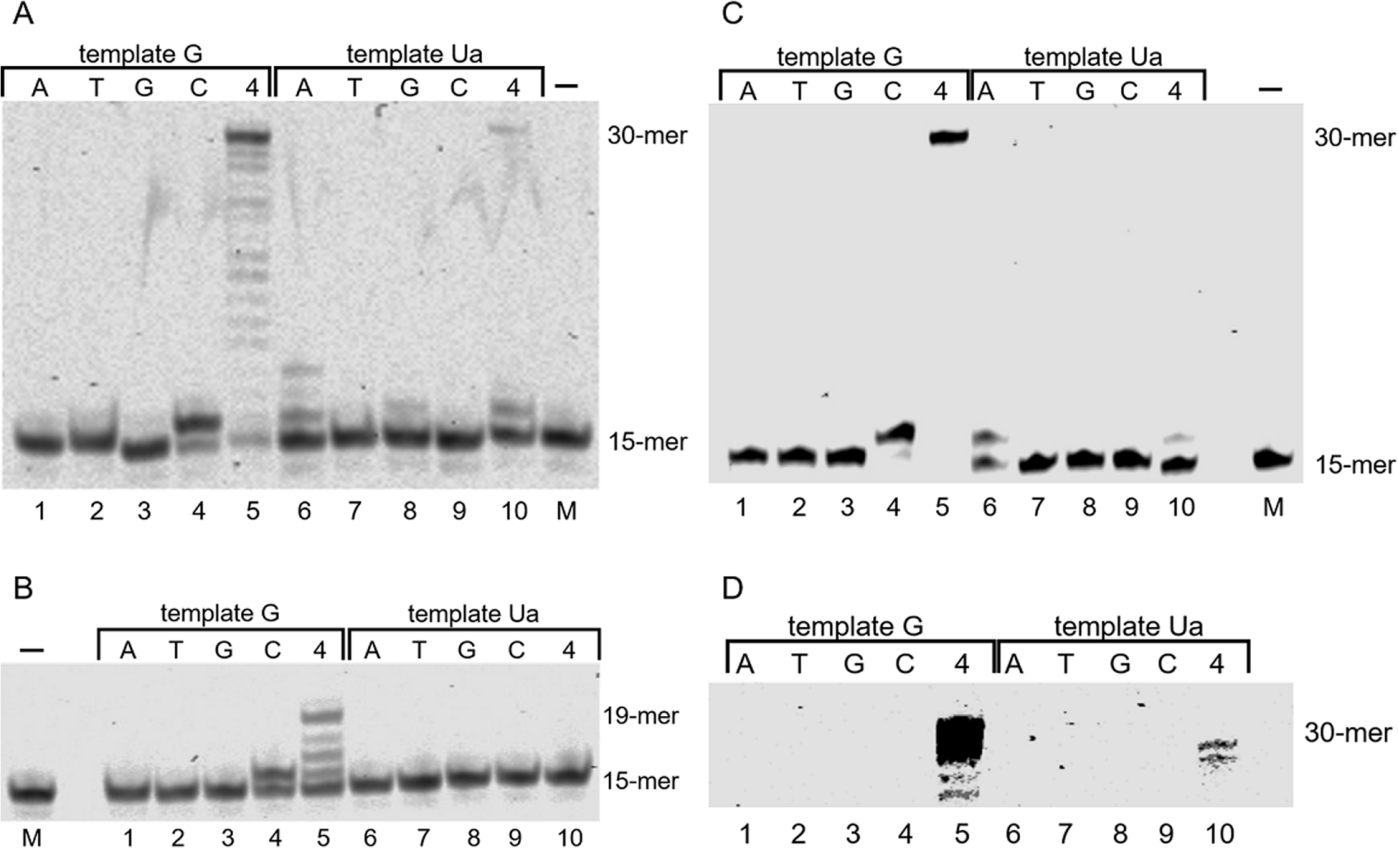
DNA synthesis and selective nucleotide incorporation opposite urea (Ua) by DNA polymerase α (panel A), DNA polymerase β (panel B) and Kf exo^−^ (panels C and D). DNA polymerase α (100 μU), DNA polymerase β (250 μU) and Kf exo^−^ (75 μU) ware incubated with templates containing G (lanes 1–5) or Ua (lanes 6–10) and 100 μM of each of the four dNTPs (lanes 5 and 10) or 100 μM of a single dNTP (N = C, G, A, or T) (lanes 1–4 and 6–9). Lane M contained no enzyme and are negative controls. The background darkness of panel C has been adjusted in panel D, and only the part of the gel around the 30-mer full length products is shown

DNA polymerase α was used for in vitro nucleotide insertion and the analysis of primer extension of template oligonucleotides containing Ua. Under the reaction condition in which DNA polymerase α inserted only dCTP opposite guanine (Fig. 2A, lanes 1–4), dATP and dGTP were inserted opposite Ua, and dTTP and dCTP were not inserted (Fig. 2A, lanes 6–9). Lane 6 of Fig. 2A shows that one or more dATP nucleotides were inserted, with a nucleotide incorporation ratio of 21%. The yield of dGTP insertion opposite Ua was 6% (Fig. 2A, lane 8). These results show that dATP, and not dGTP, was predominantly inserted opposite Ua by DNA polymerase α.

We investigated whether DNA polymerase α extends the primer beyond Ua. DNA polymerase α slightly elongated the 15-mer primer to 30-mer (3%) across the Ua lesion (Fig. 2A, lane 10), although the primer extension across Ua was of low efficiency as compared with primer extension across guanine (87%) (Fig. 2A, lanes 5 and 10).

DNA polymerase β could not insert any nucleotide opposite Ua under the condition that accurately inserted dCTP opposite guanine (Fig. 2B, lanes 1–4 and 6–9). On the one hand, Kf exo^−^ inserted dATP opposite Ua, whereas dTTP, dGTP, and dCTP were not inserted (Fig. 2C, lanes 6–9) under the condition that inserted only dCTP opposite guanine (Fig. 2C, lanes 1–4). Thus, Kf exo^−^ incorporated one nucleotide, then further incorporations were largely stalled (Fig. 2D, lane 10, and Fig. S2).

**Fig. 3:**
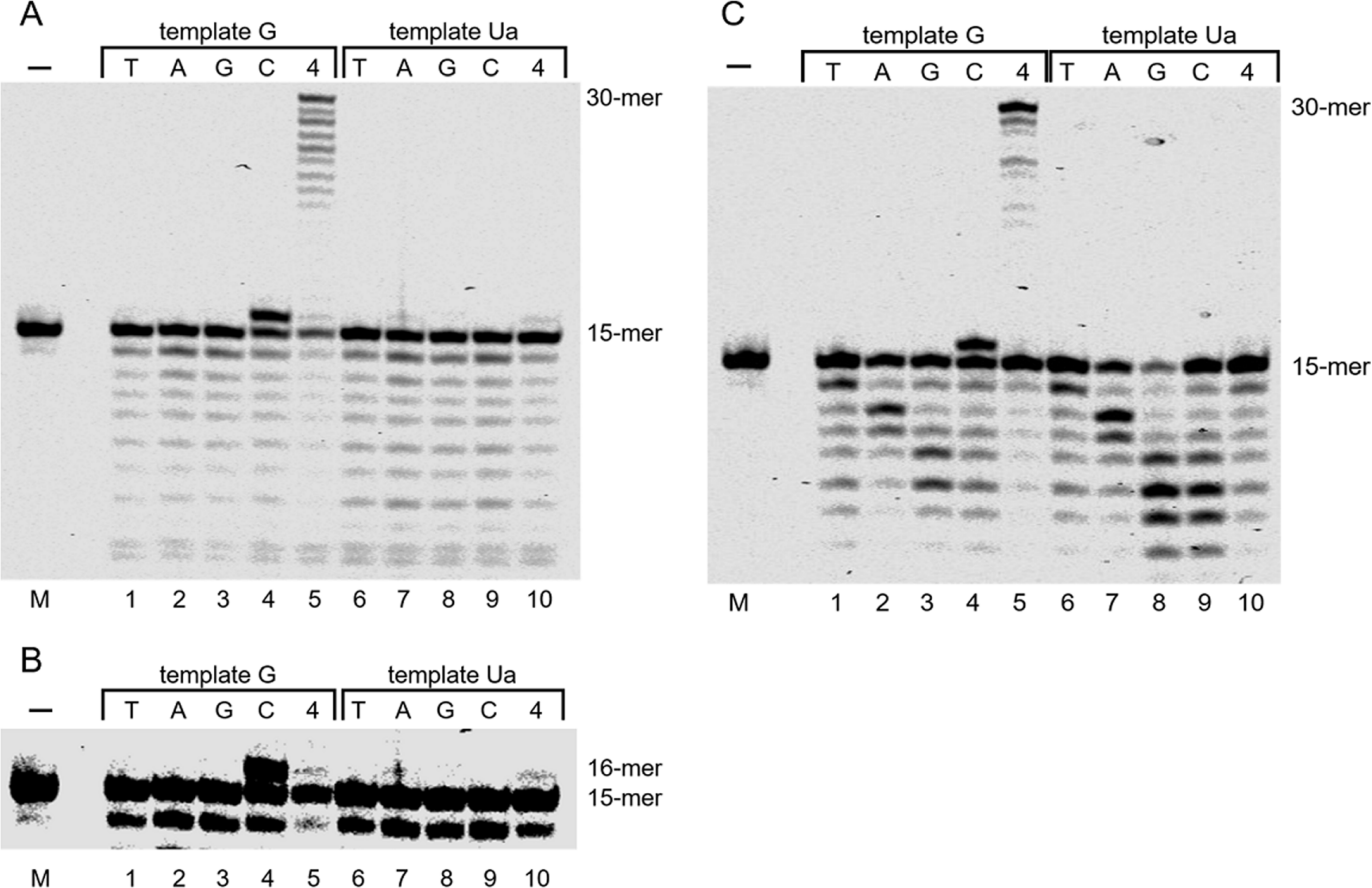
DNA synthesis and selective nucleotide incorporation opposite urea (Ua) by DNA polymerase δ (panels A and B) and DNA polymerase ε (panel C). DNA polymerase δ (154 ng) and DNA polymerase ε (50 μU) ware incubated with templates containing G (lanes 1–5) or Ua (lanes 6–10) and 100 μM of each of the four dNTPs (lanes 5 and 10) or 100 μM of a single dNTP (N = C, G, A, or T) (lanes 1–4 and 6–9). Lane M contained no enzyme and are negative controls. The background darkness of panel A has been adjusted in panel B, and only the part of the gel around the 30-mer full length products is shown

DNA polymerase δ inserted only dATP (7%) opposite Ua under the condition that incorporated only dCTP into guanine in the template (Fig. 3A, lane 7, and Fig. 3B, lane 7). In contrast, DNA polymerase ε did not insert any nucleotide (Fig. 3C, lanes 6–9). DNA polymerase δ did not elongate the 15-mer primer after dATP was inserted opposite Ua, and DNA polymerase ε did not elongate the primer even in the presence of all four dNTPs.

### Incorporation and translesion synthesis opposite Ua by translesion synthesis DNA polymerases

**Fig. 4:**
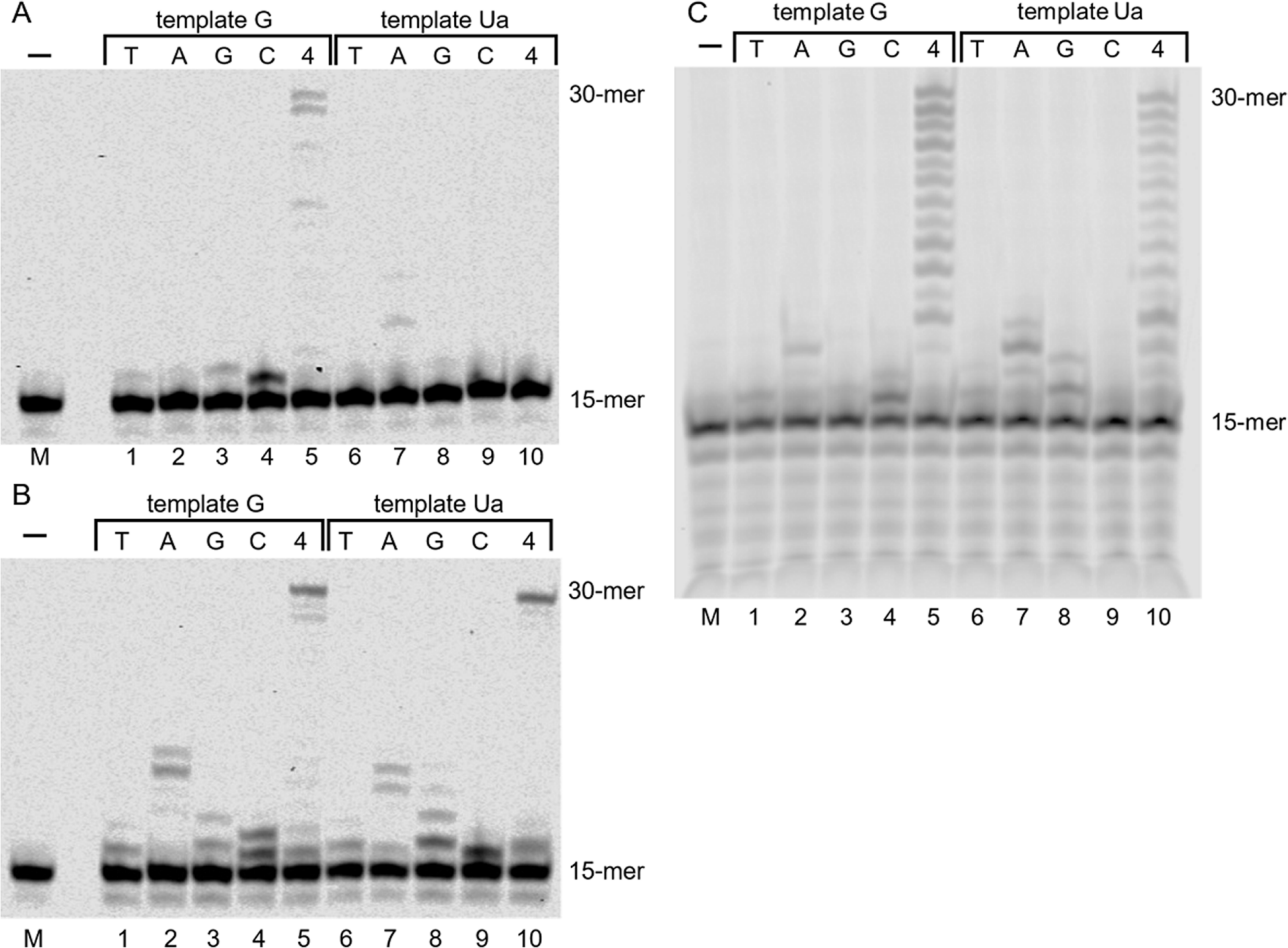
DNA synthesis and selective nucleotide incorporation opposite urea (Ua) by DNA polymerase ζ (panel A), DNA polymerase η (panel B) and *Sulfolobus solfataricus* P2 DNA polymerase IV (Dpo4) (panel C). DNA polymerase ζ (1.7 ng), DNA polymerase η (0.4 ng) and Dpo4 (0.4 ng) ware incubated with templates containing G (lanes 1–5) or Ua (lanes 6–10) and 100 μM of each of the four dNTPs (lanes 5 and 10) or 100 μM of a single dNTP (N = C, G, A, or T) (lanes 1–4 and 6–9). Lane M contained no enzyme and are negative controls

DNA polymerase ζ predominantly inserted mainly dATP (3%) opposite Ua (Fig. 4A, lane 7) but no full-length extension across Ua was detected (Fig. 4A, lane 10). DNA polymerase η efficiently elongated the primer up to full-length past Ua (Fig. 4B, lane 10, and Fig. S3) and incorporated dCTP (25%), dGTP (21%), dATP (15%), or dTTP (10%) opposite Ua (Fig. 4B, lanes 6–9). Dpo4 inserted dGTP (16%) and dATP (14%) opposite Ua (Fig. 4C lanes 6–9) under the condition that inserted mainly dCTP opposite guanine (Fig. 4C lanes 1–4), and could elongate the primer across Ua, although the elongation efficiency beyond Ua was slightly inferior to that across guanine (Fig. 4C lanes 5 and 10).

## Discussion

In previous reports, DNA synthesis across Ua and nucleotide incorporation opposite Ua was analyzed using DNA polymerase β and Kf exo^−^ [25, 28]. Here, using DNA polymerase β and Kf exo^−^, we re-analyzed nucleotide incorporation and translesion synthesis opposite Ua. The results of elongation efficiency by DNA polymerase β beyond Ua (Fig. 2B) confirm that Ua is an impediment to DNA synthesis [28].

*Escherichia coli* DNA polymerase I lacking 3′ → 5′ exonuclease activity was previously shown to insert guanine and adenine opposite Ua [25]. In addition, it was reported that adenine is incorporated preferentially opposite Ua compared with guanine [28]. The present results (Fig. 2C) are in good agreement with previous reports.

DNA polymerases α, δ and ε are replicative DNA polymerases belonging to the B-family. DNA polymerase α is associated with DNA primase and is an enzyme that can initiate DNA synthesis. In our results, DNA polymerase α inserted dATP, but primer extension by DNA polymerase α beyond Ua was of low efficiency (Fig. 2A). Hence, DNA polymerase α does not actively participate in translesion synthesis across Ua. Moreover, DNA polymerases δ and ε are involved in lagging and leading strand synthesis [33, 34]. Unlike DNA polymerase α, DNA polymerases δ and ε have 3′ → 5′ exonuclease activity, and this activity is involved in the fidelity mechanisms of proofreading, mismatch repair, and Okazaki fragment maturation. However, DNA polymerases δ and ε did not elongate the primer up to full-length across Ua, and produced degraded products of these primers (Fig. 3). Therefore, Ua is a lesion that strongly inhibits elongation by DNA polymerases δ and ε.

DNA polymerase ζ plays a critical role in an error-prone lesion bypass pathway [35–37]. We previously reported that the elongation efficiency to full-length beyond 2,2,4-triamino-5(2*H*)-oxazolone (Oz) using DNA polymerase ζ was approximately the same as that of natural DNA [29]. Analysis of nucleotide selectivity shows that DNA polymerase ζ predominantly inserted mainly dATP opposite Ua but did not elongate the primer up to full-length across Ua (Fig. 4A). These results show that Ua has an inhibitory effect on DNA polymerase ζ.

DNA polymerase η can efficiently and accurately extend the primer beyond the cyclobutane pyrimidine dimer [38], as well as moderately elongate the primer to full length across Oz [39]. The present results show that DNA polymerase η incorporated each of the four dNTPs opposite Ua, and only dGTP incorporation was increased opposite Ua compared with guanine (Fig. 4B). In addition, full-length extension across Ua was detected (Fig. 4B). Many previous reports showed that DNA polymerase η elongated primers across many DNA lesions [38–43]. Similarly, DNA polymerase η elongated the primer across Ua.

**Fig. 5:**
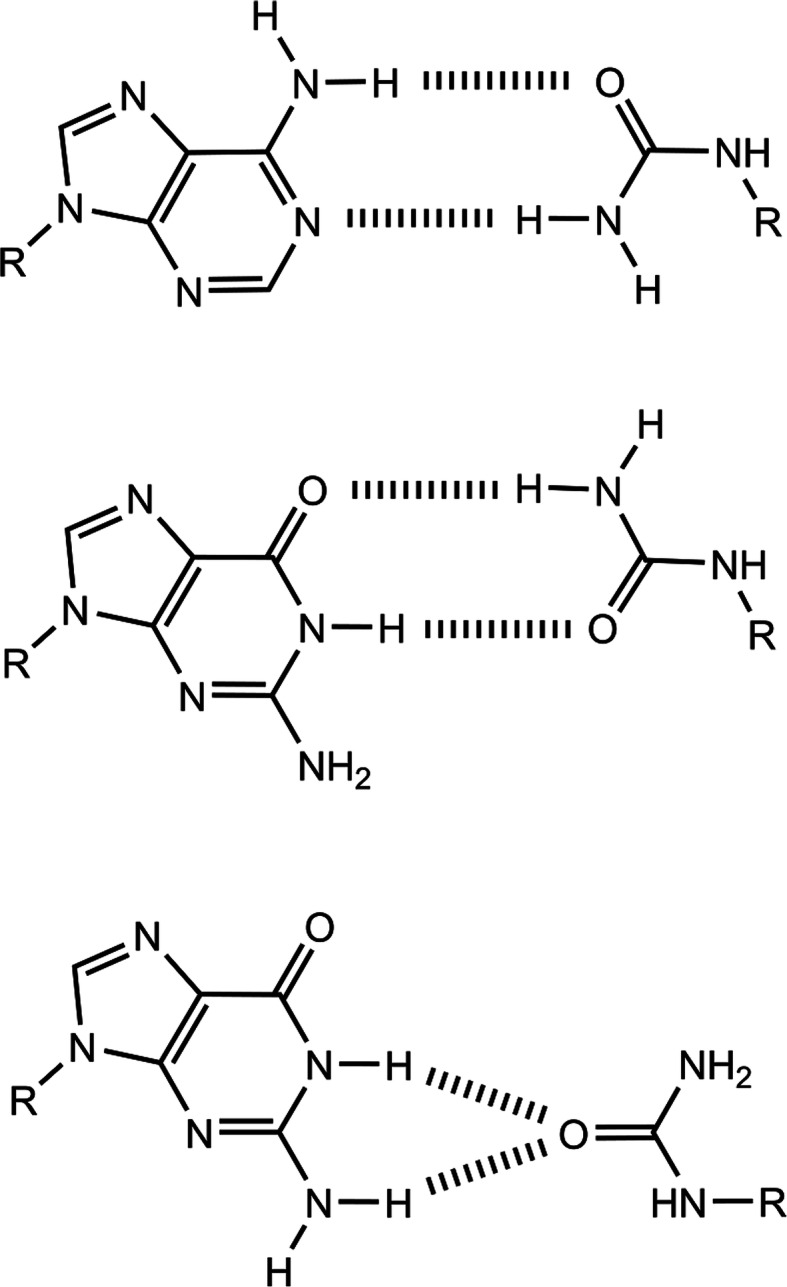
Possible urea (Ua):A and Ua:G base pairs obtained from molecular dynamics simulations [45]

Dpo4 is a thermostable translesion synthesis polymerase and elongated the primer to full-length across Ua (Fig. 4C, lane 10). In addition, Dpo4 inserted dATP and dGTP opposite Ua to almost the same degree (Fig. 4C, lanes 6–9). In a previous report [44], Dpo4 inserted dATP opposite an abasic site more efficiently than dGTP. This difference may be attributed to forming base pairs via hydrogen bonds, as shown in Fig. 5 [45].

## Conclusion

DNA polymerases α, δ, ζ, and Kf exo^−^ predominantly inserted dATP opposite Ua, whereas DNA polymerase η inserted dCTP, dGTP, dATP and dTTP. DNA polymerases β and ε were blocked by Ua, whereas polymerase α and Kf exo^−^ slightly elongated the primer to full-length across Ua. Elongation by DNA polymerases δ and ζ stopped after dATP was inserted opposite Ua. Surprisingly, DNA polymerase η and Dpo4 expanded the primer to full-length across Ua, similar to across guanine. Therefore, the stalling of DNA replication caused by the generation of Ua can be avoided using DNA polymerase η and Dpo4. In the future, reactions of other DNA polymerases and templates containing Ua will be investigated.

## Supplementary Information

**Additional file 1: Fig. S1.** Construction of the 30-merUa. Two products (30-merUa1 and 30-merUa2) were obtained (Materials and methods). However, these two products equilibrate with each other and thus could not be isolated separately. In a previous report [7], Dubey, et al. revealed that Ua comprises the α- and β-anomers. The mixture of the two products (C_289_H_370_N_103_O_176_P_29_) was confirmed by ESI-MS (m/z 9000.727) and then was used as 30-merUa in our experiment.

**Additional file 2: Fig. S2.** DNA synthesis across urea (Ua) by Kf exo^−^. DNA synthesis in **Fig. S2** was conducted under the same condition as in Fig. 2C. Kf exo^−^ (75 μU) was incubated with templates containing G (lane 3) or Ua (lane 4) and 100 μM of each of the four dNTPs (lanes 1–4). Lanes 1 and 2 contained no enzyme and are negative controls. The background darkness of panel A is adjusted in Panel B. **Fig. S3.** DNA synthesis across urea (Ua) by DNA polymerase η. DNA synthesis in **Fig. S3** was conducted under the same condition as in Fig. 4B. DNA polymerase η (0.4 ng) was incubated with templates containing G (lane 2) or Ua (lane 4) and 100 μM of each of the four dNTPs (lanes 1, 2, 4 and 5). Lanes 1 and 5 contained no enzyme and are negative controls. The sample in lane 3 was a mixture of the samples in lanes 2 and 4.

## Data Availability

All data generated during this study are included in this published article.

## Abbreviations

Ua: Urea
Kf: Klenow fragment
Dpo4: *Sulfolobus solfataricus* P2 DNA Polymerase IV
30-merUa: 30-mer DNA template containing Ua
6-merUa: 6-mer oligonucleotide containing Ua
Oxa: Oxaluric acid
BSA: Bovine serum albumin
Oz: 2,2,4-triamino-5(2*H*)-oxazolone
