# Supplementary material for: Analysis of nucleotide insertion opposite urea and translesion synthesis across urea by DNA polymerases
Source: Genes Environ. 2022 Feb 15;44:7. doi: 10.1186/s41021-022-00236-3 (PMC8845263; doi:10.1186/s41021-022-00236-3)
Supplement: Supplementary file 1 — Additional file 1: Fig. S1. Construction of the 30-merUa. Two products (30-merUa1 and 30-merUa2) were obtained (Materials and methods). However, these two products equilibrate with each other and thus could not be isolated separately. In a previous report [7], Dubey, et al. revealed that Ua comprises the α- and β-anomers. The mixture of the two products (C289H370N103O176P29) was confirmed by ESI-MS (m/z 9000.727) and then was used as 30-merUa in our experiment. [file 41021_2022_236_MOESM1_ESM.docx]

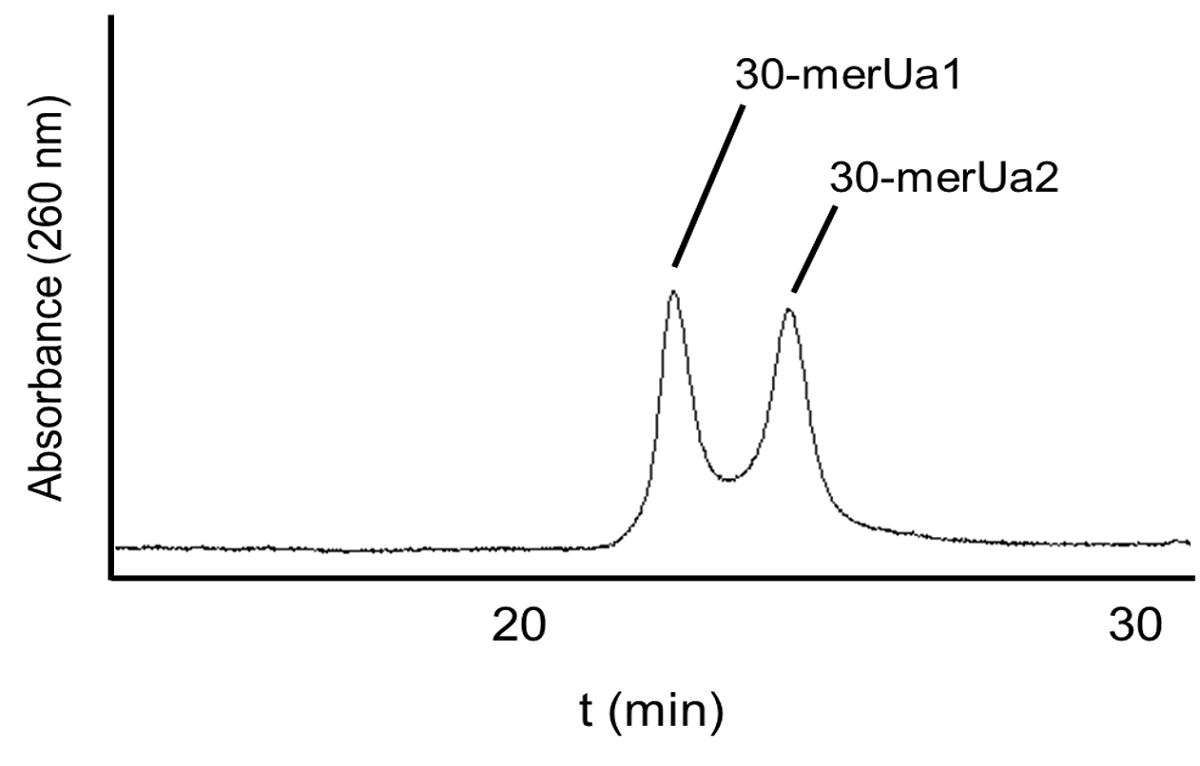


Figure S1. Construction of the 30-merUa. Two products (30-merUa1 and 30-merUa2) were obtained (Materials and methods). However, these two products equilibrate with each other and thus could not be isolated separately. In a previous report (7), Dubey, *et al*. revealed that Ua comprises the α- and β-anomers. The mixture of the two products (C_289_H_370_N_103_O_176_P_29_) was confirmed by ESI-MS (m/z 9000.727) and then was used as 30-merUa in our experiment.
