# Supplementary material for: Analysis of nucleotide insertion opposite urea and translesion synthesis across urea by DNA polymerases
Source: Genes Environ. 2022 Feb 15;44:7. doi: 10.1186/s41021-022-00236-3 (PMC8845263; doi:10.1186/s41021-022-00236-3)
Supplement: Supplementary file 2 — Additional file 2: Fig. S2. DNA synthesis across urea (Ua) by Kf exo−. DNA synthesis in Fig. S2 was conducted under the same condition as in Fig. 2C. Kf exo− (75 μU) was incubated with templates containing G (lane 3) or Ua (lane 4) and 100 μM of each of the four dNTPs (lanes 1–4). Lanes 1 and 2 contained no enzyme and are negative controls. The background darkness of panel A is adjusted in Panel B. Fig. S3. DNA synthesis across urea (Ua) by DNA polymerase η. DNA synthesis in Fig. S3 was conducted under the same condition as in Fig. 4B. DNA polymerase η (0.4 ng) was incubated with templates containing G (lane 2) or Ua (lane 4) and 100 μM of each of the four dNTPs (lanes 1, 2, 4 and 5). Lanes 1 and 5 contained no enzyme and are negative controls. The sample in lane 3 was a mixture of the samples in lanes 2 and 4. [file 41021_2022_236_MOESM2_ESM.docx]

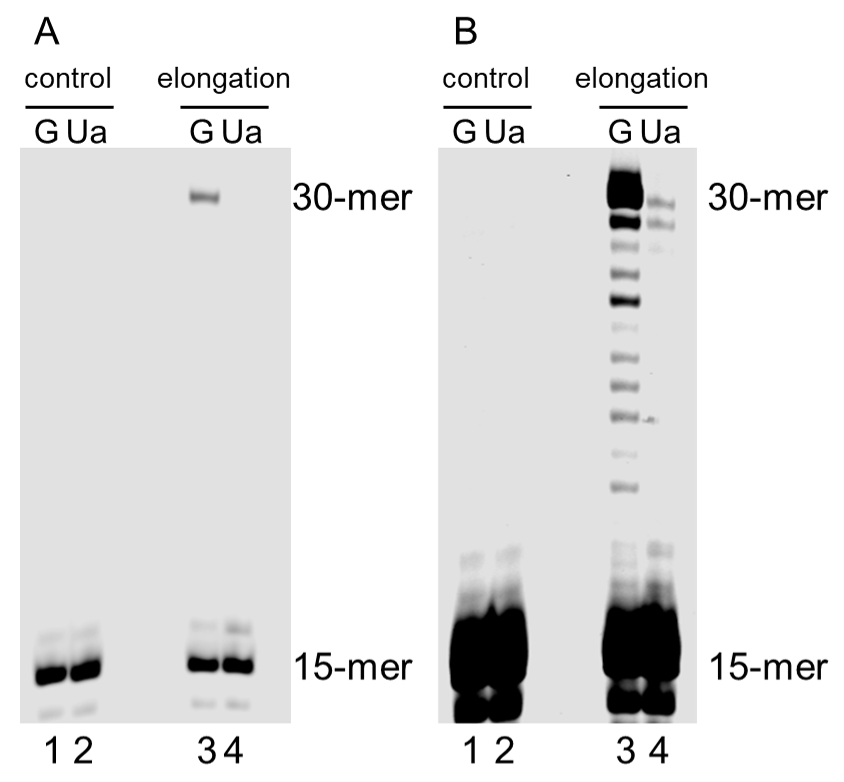


Figure S2. DNA synthesis across urea (Ua) by Kf exo^-^. DNA synthesis in Figure S1 was conducted under the same condition as in Figure 2C. Kf exo^-^ (75 μU) was incubated with templates containing G (lane 3) or Ua (lane 4) and 100 μM of each of the four dNTPs (lanes 1-4). Lanes 1 and 2 contained no enzyme and are negative controls. The background darkness of panel A is adjusted in Panel B.


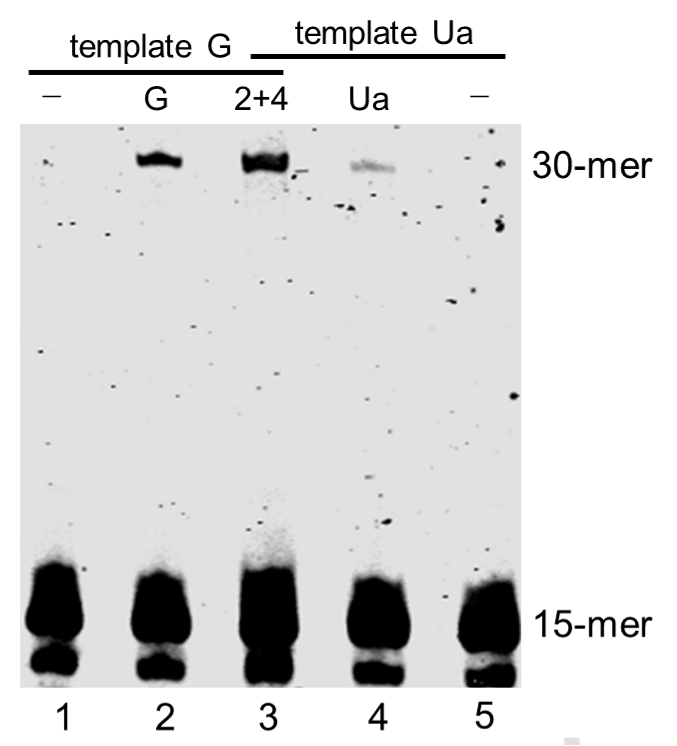


Figure S3.

DNA synthesis across urea (Ua) by DNA polymerase η. DNA synthesis in Figure S2 was conducted under the same condition as in Figure 4B. DNA polymerase η (0.4 ng) was incubated with templates containing G (lane 2) or Ua (lane 4) and 100 μM of each of the four dNTPs (lanes 1, 2, 4 and 5). Lanes 1 and 5 contained no enzyme and are negative controls. The sample in lane 3 was a mixture of the samples in lanes 2 and 4.
